# Determination of haematological and biochemical parameters of Calf Saiga antelope (*Saiga tatarica*) living in the Gansu Endangered Animals Research Center

**DOI:** 10.1002/vms3.236

**Published:** 2020-01-25

**Authors:** Xiaohua Du, Xia Liu, James Blackar Mawolo, Haifang Wang, Xiaoyu Mi, Jianying Dong, Qiao Li, Yongqiang Wen

**Affiliations:** ^1^ College of Veterinary Medicine Gansu Agricultural University Lanzhou City Gansu Province People’s Republic of China; ^2^ College of Life Science and Technology Gansu Agricultural University Lanzhou City Gansu Province People’s Republic of China

**Keywords:** biochemical parameters, Calf Saiga antelope, haematological, homeothermy and Gansu Province, reference ranges

## Abstract

**Background:**

The Saiga antelope (*Saiga tatarica*) is native to Eurasia and is a member of the family Bovidae. Prior to 1920, the antelope had been extensively hunted for its horns, which were used in traditional Chinese medicine. Since 1920, the Saiga antelope has been protected because of this extensive hunting, which nearly led to its extinction.

**Objective:**

The study evaluated haematological and biochemical parameters to provide references for the Calf Saiga antelope (*S. tatarica*). The study also sought to explore the mechanisms affecting these parameters in both genders of the Calf Saiga antelope.

**Methods:**

Haematological and biochemical parameters were collected from the Calf Saiga antelope. Haematological and biochemical parameters were analysed by the Coulter counter and Automatic analyser, respectively.

**Results:**

The average concentrations of female triglyceride levels showed significantly higher values than the significant concentrations of male. Female red blood cells and platelets concentrations were statistically significant than the significant concentrations of males. Magnesium female concentrations were also significantly higher than male values. Other parameters showed differences between males and females.

**Conclusion:**

The reported results show that haematological and biochemical characteristics varied among Calf Saiga antelope and other animals. The study results suggest that regardless of the factors, breed, the breeding environment, and climatic variables, haematological and biochemical variations can be triggered that can result in a reduction in the heat production needed for maintenance of homeothermy.

## Graphical abstract

The study assessed reference values of the Calf Saiga antelope living in Gansu Province. The findings indicated that parameters of Calf Saiga antelope differ from other members of the Bovidae family.

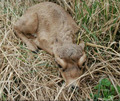

## 1 INTRODUCTION

Abundant data with reference ranges of both domestic and Przewalski's horses (Plotka, Eagle, Gaulke, Tester, & Siniff, 1988; Tomenendalova, Vodicka, Uhrikova, & Doubek, 2014), beef cow, Catalonian donkeys (*Equus asinus*), and goats and sheep, both the species and individual breeds (Alberghina et al., 2010; Folch, Jordana, & Cuenca, 1997; Giuseppe, Augusto, Cristiano, & Giovanni, 2006) exist. Reference ranges of biochemical and physiological parameters can be useful parameters for evaluating the state of health in different specimens of each species in addition to diagnosing and preventing diseases (Bezerra et al., 2008). On the contrary, biochemical and physiological reference ranges of Calf Saiga antelope are lacking. Saiga antelope (*S. tatarica*) is a member of the family Bovidae and is native to Eurasia. Because of its near extinction due to hunting, the animal has been protected since 1920. Saiga antelope were hunted for their horns, which were used in traditional Chinese medicine. Due to its near extinction, Saiga antelope is one of the few species classified as endangered. The species was saved by captive breeding, which led to its successful return that was limited in the wild. However, concerns about the lives of Calf Saiga antelope are alarming after multiple antelope generations. Up to present, no data or reports about the Calf Saiga antelope exist. Recently in 2015, over 200,000 Saiga antelope, half of the world's Saiga antelope population, died from a suspected bacterial infection called Pasteurellosis, caused by the bacterium Pasteurella multocida type B, which results in haemorrhagic septicaemia (Nicholls, 2015). Calf Saiga antelope are breastfed by their mothers for up to 4 months. In captivity, Calf Saiga antelope are nursed by other nonrelated adult Saiga antelopes. At birth, the weight of the Calf Saiga antelope is 3.5 kg, and they grow to an average of 14.5 kg during weaning. Calf Saiga antelope living in the centre for endangered antelopes live under similar conditions. Therefore, climatic region and their fitness and states of health should be homogeneous. Furthermore, blood parameters can be influenced by the sampling procedure, anaesthesia, immobilization, breed, sex, age, nutrition, diseases, handling and stress.

## 2 MATERIALS AND METHODS

All experimental procedures used in this study were approved by Animal Ethical and Welfare Committee of Gansu Endangered Animals Research Center, Gansu Province in September 2018. Approval No. AEWC‐GEARC‐2018006.

### 2.1 Animals

A total of 25 and 36 healthy male and female Calf Saiga antelopes, respectively, were used in this study. The age range of the Calf Saiga antelope included in this study was 1–3 days old. Calf Saiga antelopes involved in this study belong to the Gansu Endangered Animals Research Center, located in Wuwei city of Gansu Province, People's Republic of China. Presently, over 100 Saiga antelope were living at the centre. The Gansu Endangered Animals Research Center was established in 1987 and covers a land area of 180, 000 hectares.

### 2.2 Specimen collection

Calf Saiga antelope were breastfed by their mothers prior to blood sample collection. During blood sample collection, Calf Saiga antelopes were immobilized (minimally). Samples were then collected on separate occasions. Biochemical parameters were collected within 3 days, whereas physiological parameters were collected in 2 days. Specimens were collected during early morning and evening hours. Blood samples were collected from the jugular vein of the animals into vacuette test tubes (6 ml) containing potassium and disodium ethylenediaminetetraacetic acid (K_2_/Na_2_‐EDTA). Afterwards, biochemical analyses were done within five hours after blood collection using an automated analyser (Hitachi Ltd 7,180 Serial, Tokyo, Japan) at the Animal Biochemistry Laboratory of the Science and Technology College of Gansu Agricultural University. During the analysis of biochemical parameters, blood samples were centrifuged at 1,000 rpm for 10 min. Serum and blood plasma were transferred into polypropylene microcentrifuge test tubes, which were stored at –80°C until analysis. After the collection of physiological parameters, blood samples were check for five hours using a Coulter counter (Model ZK). All test tubes were sterilized prior to use. Every effort was made to minimize both the number of animals used and animal suffering during the experiments.

### 2.3 Laboratory examination

Red and white blood cell counts were obtained using the Coulter counter method. Red blood cells were stained with Dapi's solution (Florida, USA). A quantity of 25 μl of blood were added to vials containing 4,975 μl of Dapi's solution (200 × dilution). Red blood cells were then counted in the chamber of coulter counter with the aid of light microscope. White blood cells (WBC) were counted using Neubaer's solution (Berlin's Germany). A 25 μl aliquot of blood was added to vials containing 475 μl of Naubaer's solution (20 × dilution). White blood cell differential counts were obtained from blood smears stained with Diff‐Quick and counted under an oil immersion objective and 1000x magnification. Determination of lymphocyte counts was done using complete blood count test. While absolute neutrophil count (ANC) was used to determine granulocyte and platelets, granulocytes were counted as part of the white blood cell differential test.

Haematocrit and intermediates were obtained following blood centrifugation in microcapillaries, whereas haemoglobin concentrations were measured using the Coulter method (England). Biochemical parameters (urea and creatinine kinase) were determined using spectrophotometry. Glucose, total protein, cholesterol, alkaline phosphatase (ALP) and hydroxybutyrate dehydrogenase (HBDH) concentrations were measured using BioVendor (Czech Republic) tests. Quantitative serum tests were used to determine immunoglobulin. The albuimin concentrations were determined using the microalbumin test (Beijing, People Republic of China). The Phosphate Test (HUMAN Diagnostics, Germany) was used for the determination of alkaline phosphatase concentrations, triglycerides and inorganic phosphorus.

Concentrations of minerals, such as calcium and magnesium, were determined using spectrophotometric methods.

### 2.4 Statistical analysis

Results are described using means and standard deviation, medians, minimum, maximum and number of samples. Outliers were excluded using Grubbs’ test (Lumsden & Mullen, 1978). Additionally, the means and standard deviation were compared with published results (Baronetzky‐Mercier, 1992; Kuttner & Wiesner, 1987) and the gender differences were compared using the *F*‐test for homogeneity of variances and the *t*‐test. Values of *p* < .05 and *p* < .01 were considered statistically significant and highly statistically significant, respectively. MS Excel was used to perform statistical analyses.

## 3 RESULTS

### 3.1 Haematological parameters

Table 1 presents the range of haematological parameters. All specimens were evaluated without consideration of gender. In this table, haemoglobin shows higher value, whereas granulocytes read lower value. Other parameters fell below and within the reference range. Meanwhile, Table 2 shows a comparison of males and females (means and standard deviations in addition to minimum and maximum values) parameters. In Table 2, red blood cell, platelet, intermediate and granulocyte counts showed statistically significant differences. Statistical significance was evaluated using the *t*‐test. Table 3 shows a comparison of haematological parameters based on all examined specimens (without considering gender) with published data (Baronetzky‐Mercier, 1992; Kuttner & Wiesner, 1987; Lumsden, Rowe, & Mullen, 1980). In this table, our results were somewhat similar to those previously reported.

**Table 1:** Range of haematological parameters

Variables	*x* ± *SD*	Median	Min–max	*n*
RBC (×10^12^/L)	6.6 ± 1.2	6.32	4.92–9.82	21/35
Haematocrit (l/l)	0.41 ± 0.05	0.42	0.3–0.52	23/37
Haemoglobin (g/l)	132.07 ± 22.14	134.75	91.55–187.15	17/32
WBC (×10^9^/L)	6.68 ± 1.55	6.15	4.3–10.2	21/36
Platelets (×10^9^/L)	0.26 ± 0.14	0.28	0–0.73	21/35
Intermediate (×10^9^/L)	3.08 ± 1.05	2.85	1.57–7.24	21/36
Lymphocytes (×10^9^/L)	2.92 ± 0.80	2.86	1.68–4.84	21/36
Granulocytes (×10^9^/L)	0.09 ± 0.16	0	0–0.62	21/36

Note

Haemoglobin shows higher value, whereas granulocytes read lower value. Other parameters were below and within the reference range. All specimens were evaluated without consideration of gender.

Abbreviations: Min, minimum and Max, maximum values, *n*, number of Calf/samples; *SD* = standard deviation.

**Table 2:** Comparison of males and females haematological parameters

Variables	Male			Female			Statistics	
	*x* ± *SD*	Min‐Max	*n*	*x* ± *SD*	Min‐Max	*n*	*F*‐test	T‐test
RBC (×10^12^/L)	6.26 ± 0.8	5.00–8.22	14/22	7.19 ± 1.54	4.92–9.82	7/13	0.008**	0.031*
Haematocrit (l/l)	0.41 ± 0.05	0.30–0.52	15/24	0.42 ± 0.05	0.34–0.51	7/13	0.535	0.242
Haemoglobin (g/l)	128.0 ± 20.20	98.20–160.70	13/21	139.84 ± 24.46	91.55–187.15	5/11	0.453	0.077
WBC (×10^9^/L)	6.42 ± 1.51	4.30–10.00	15/24	7.22 ± 1.56	4.90–10.22	6/12	0.849	0.074
Platelets (×10^9^/L)	0.23 ± 0.16	0.00–0.73	15/24	0.33 ± 0.07	0.20–0.44	6/12	0.018	0.019*
Intermediate (×10^9^/L)	2.96 ± 0.76	1.87–4.59	15/24	3.31 ± 1.48	1.57–7.24	6/13	0.007**	0.231
Lymphocytes (×10^9^/L)	2.86 ± 0.76	1.68–4.60	15/24	3.02 ± 0.90	2.04–4.84	6/13	0.476	0.299
Granulocytes (×10^9^/L)	0.06 ± 0.11	0.00–0.39	15/24	0.16 ± 0.22	0.00–0.62	6/11	0.007**	0.102

Abbreviations: Min, Minimum and Max, maximum values, *n*, number of Calf/samples; *SD*, standard deviation.

* *p* < .05.

** *p* < .01. Red blood cell, platelets, intermediate and granulocyte showed statistical significance, whereas other parameters were below and above the reference range.

**Table 3:** Comparison of haematological parameters with published results about the Przewalski and domestic horses

Variables	Calf Saiga antelope (*n* = 30–37)	Equus Przewalski (*n* = 20)	E.p. ISIS (*n* = 39–78)	Ergebinese dei (*n* = 4)	Equus horse (*n* = 4)	Light horse (*n* = 44–50)
RBC (×10^12^/L)	6.60 ± 1.70	8.9 ± 0.9	NA	NA	8.6 (8.4–10.8)	8.8 ± 0.95
Haematocrit (l/l)	73.59 ± 13.32	43.7 ± 3.7	0.42 ± 0.07	NA	0.47 (0.38–0.50)	0.40 ± 0.04
Haemoglobin (g/l)	151.33 ± 42.63	155 ± 1.7	154 ± 2	NA	163 (14.3–20)	146 ± 16.5
WBC (×10^9^/L)	2.48 ± 0.91	8.26 ± 1.68	8.3 ± 2.5	7.5 (5.1–9.2)	9.6 (8.4–10.8)	7.45 ± 1.28
Platelets (×10^9^/L)	0.26 ± 0.14	NA	NA	NA	NA	0.06 ± 0.1
Intermediate (×10^9^/L)	3.08 ± 1.05	5.18 ± 1.44	NA	NA	NA	3.75 ± 0.88
Lymphocytes (×10^9^/L)	0.49 ± 0.24	2.8 ± 0.81	154 ± 2	NA	NA	3.10 ± 0.75
Granulocytes (×10^9^/L)	0.09 ± 0.16	NA	NA	NA	NA	0.2 ± 0.2

Abbreviations: NA, not available, *n*, number of samples, in this table, our results were slightly similar to what was reported by other researchers.

### 3.2 Biochemical parameters

Table 4 presents range of biochemical parameters. Specimens were evaluated without consideration of gender. In this table, Creatinine kinase shows highest value without significant difference, whereas immunoglobulin was the lowest value. Other parameters were below and within the reference range. Table 5 reports that triglyceride level presents statistically significant differences, whereas Table 6 compares the results of this study with published data (Baronetzky‐Mercier, 1992; Kuttner & Wiesner, 1987). In Table 6, the reported results were nearly similar to other published data.

**Table 4:** Ranges of biochemical parameters

Variables	*x* ± *SD*	Median	Min–max	*n*
Total protein (g/L)	64.1 ± 7.2	65.5	48.9–81	24/41
Albumin (g/L)	32 ± 3.5	32.1	24.5–38.5	24/41
Glucose (mmol/L)	8.68 ± 2.77	8.35	4.01–15.07	24/41
Creatinine (μmol/L)	121.67 ± 18.53	120.2	78.3–157.7	24/41
Urea (mmol/L)	5.89 ± 1.85	5.3	2.93–9.92	24/41
Triglyceride (μmol/L)	17.7 ± 7.9	15.95	6.6–37.7	24/40
ALP (μkat/L)	3.53 ± 0.92	3.37	1.89–5.59	24/41
Immunoglobin (μkat/L)	0.27 ± 0.08	0.26	0.15–0.51	24/41
CK (μkat/L)	4.97 ± 1.99	4.57	2.22–9.76	21/37
HBDH (μkat/L)	14.79 ± 2.75	14.54	10.64–20.26	22/38
Cholesterol (mmol/L)	1.83 ± 0.32	1.77	1.27–2.57	24/40
Ca (mmol/L)	2.64 ± 0.21	2.65	2.24–3.1	24/41
P (mmol/L)	1.16 ± 0.38	1.05	0.47–1.96	23/40
Mg (mmol/L)	0.64 ± 0.08	0.65	0.47–0.82	22/37

Note

Creatinine has high value, whereas immunoglobin read low value. Other parameters fell below and within the reference range. All specimens were evaluated without consideration of gender.

Abbreviations: Min, Minimum and Max, maximum values; *n*, number of Calf/samples; *SD*, standard deviation.

**Table 5:** Comparison of males and females biochemical parameters

Variables	Male			Female			Statistics	
	*x* ± *SD*	Min‐Max	*n*	*x* ± *SD*	Min‐Max	*n*	*F*‐test	T‐test
Total protein (g/L)	63.8 ± 8	48.9–81	17/27	64.7 ± 5.57	57.5–5.0.57	7/14	0.172	0.342
Albumin (g/L)	32.3 ± 3.79	24.5–38.5	17/27	31.74 ± 2.9	27.4–35.8	7/14	0.315	0.347
Glucose (mmol/L)	9.15 ± 2.93	4.45–15.1	17/24	7.76 ± 2.22	4.01–12.78	7/14	0.296	0.065
Creatinine (μmol/L)	125 ± 17.4	91.4–158	17/27	115.2 ± 19.6	78.3–148.8	7/14	0.578	0.053
Urea (mmol/L)	6.06 ± 2.08	2.93–9.92	17/27	5.55 ± 1.33	3.57–7.81	7/14	0.093	0.207
Triglyceride (μmol/L)	16.2 ± 6.86	6.6–28.7	17/26	20.57 ± 9.12	8.5–37.7	7/14	0.214	0.046*
ALP (μkat/L)	3.16 ± 0.98	1.89–5.59	17/27	3.38 ± 0.83	2.05–4.81	7/14	0.541	0.235
Immunoglobin (μkat/L)	0.24 ± 0.05	0.15–0.36	17/27	0.34 ± 0.08	0.21–0.51	7/14	0.127	2.14x10^−5**^
CK (μkat/L)	5.3 ± 1.88	2.89–9.76	15/23	4.42 ± 2.11	2.22–9.41	7/14	0.613	0.099
HBDH (μkat/L)	15.9 ± 2.69	10.6–20.3	16/25	12.69 ± 1.38	10.68–15.47	7/13	0.020*	1.17x10^−5**^
Cholesterol (mmol/L)	1.97 ± 0.29	1.44–2.57	17/27	1.55 ± 0.17	1.27–1.87	7/13	0.051	7.19x10^−7**^
Ca (mmol/L)	2.64 ± 0.23	2.23–3.1	17/27	2.63 ± 0.18	2.24–2.93	7/14	0.282	0.442
P (mmol/L)	1.15 ± 0.37	0.47–1.91	17/27	1.19 ± 0.40	0.75–1.96	6/13	0.756	0.400
Mg (mmol/L)	0.62 ± 0.09	0.47–0.82	15/24	0.67 ± 0.05	0.58–0.8	7/13	0.063	0.045*

Abbreviations: Min, Minimum and Max, maximum values; *n*, number of Calf/samples; *SD*, standard deviation.

* *p* < .05.

** *p* < .01. triglyceride shows statistically significant difference, whereas other parameters were within and below the normal range.

**Table 6:** Comparison of biochemical parameters with published results about the Przewalski and domestic horses

Variables	Calf Saiga abtelope (*n* = 22–41)	Equus Przewalski (*n* = 20)	Ergebinese dei (*n* = 39–78)	Equus horse (*n* = 4)	Light horse (*n* = 4)
Total protein (g/L)	39.218 ± 8.123	69 ± 1	64 ± 6	71 (64–81)	64 ± 5
Albumin (g/L)	23.633 ± 6.676	NA	33 ± 3	36 (33–37)	31 ± 2
Glucose (mmol/L)	7.446 ± 2.916	6.22 ± 1.17	8.27 ± 3.4	NA	4.55 ± 0.52
Creatinine (μmol/L)	501.639 ± 225.249	99.01 ± 25.64	106.01 ± 17.68	141.44–265.2	115 ± 18
Urea (mmol/L)	7.446 ± 2.916	4.13 ± 2.75	5.92 ± 1.35	4.9–11.32	NA
Triglyceride (μmol/L)	17.7 ± 7.9	16.76 ± 6.16	23.94 ± 3.42	*N*	32.5 ± 8.6
ALP (μkat/L)	3.53 ± 0.92	5.92 ± 2.22	4.15 ± 2.88	7.02–9.94	0.76 ± 0.18
Immunoglobin (μkat/L)	0.27 ± 0.08	0.28 ± 0.1	NA	0.33–0.63	NA
CK (μkat/L)	4.97 ± 1.99	2.37 ± 0.9	NA	NA	0.65 ± 0.67
HBDH (μkat/L)	14.79 ± 2.75	9.65 ± 2.35	7.82 ± 2.48	2.57–16.64	2.33 ± 0.56
Cholesterol (mmol/L)	1.027 ± 0.413	NA	2.44 ± 0.43	2.28 (2.18–2.36)	2.25 ± 0.34
Ca (mmol/L)	1.673 ± 0.135	3 ± 0.1	2.8 ± 0.23	2.38 (1.8–3.05)	2.98 ± 0.13
P (mmol/L)	1.16 ± 0.38	1.52 ± 0.42	1.55 ± 0.55	NA	1.03 ± 0.16
Mg (mmol/L)	0.203 ± 1.681	NA	NA	NA	0.82 ± 0.04

Abbreviations: NA, not available; *n* = number of samples, our results were slightly similar to what was reported by other researchers.

## 4 DISCUSSION

### 4.1 Haematological parameters of Calf Saiga antelope

Determination of haematological parameters is very important for supplying the information necessary to judge the health of an animal. Monitoring these parameters will also be useful in diagnosing and treating some animal‐related diseases (Alberghina et al., 2010). Specifically, for endangered and protected species, it is essential to be in excellent condition before being reintroduced to the wild. Gender differences were observed for haematological parameters. Males were characterized by significantly higher red blood cell counts and granulocytes (*p* < .05 in both cases) than females. Males also showed higher values of haemoglobin and white blood cells (*p* = .77 and 0.74, respectively) than females but without statistically significant differences. In addition, males showed significantly higher platelet concentrations, which is similar to studies done on horses in which platelets showed significantly different counts (Hessel, Bosch, Van Weeren, & Ionita, 2014). Contrary to this result, haemoglobin concentrations reported in the Przewalski horse were above normal values (Tomenendalova et al., 2014). This elevation resulted from the acute and intensive activities in the Przewalski horse before immobilization. Since there are limited relevant references comparing haematological parameters between genders in the Calf Saiga antelope, the authors compared the results in this study with reports on other equids. Similar differences as in the Calf Saiga antelope were reported for feral domestic horses (*E. caballus*) (Plotka et al., 1988), and males had statistically higher red blood cell counts, haematocrit values and haemoglobin concentrations when compared with females. On the contrary, researchers (Folch et al., 1997; Lumsden et al., 1980) did not find significant differences between males and females in domestic horses and Catalonian donkeys (*E. asinus*).

It is also worth noting that the levels of physiological parameters (such as red and white blood cells, haematocrit and haemoglobin) obtained in this study for the Calf Saiga antelope were lower than those reported by other authors for both domestic horses (Lumsden et al., 1980) and Saiga antelope (Hawkey, 1975; Kuttner & Wiesner, 1987). The slight differences in the ranges of the examined haematological and biochemical parameters and the reference values in domestic horses could be caused by many factors.

According to Maria, Neila, Riccrado and Roberto (2018), similar factors were shown to affect the haematological and biochemical parameters in goats and Calf Saiga antelopes in addition to other mammals. On the other hand, it is possible that differences may arise due to subclinical anaemia or adoption of different procedures for counting blood elements (manual vs. automated counters). For example, Kuttner and Wisner (1987) reported a significant decline in haemoglobin, haematocrit and red blood cell counts in Przewalski horses that underwent anaesthesia‐induced sequestration in the spleen (Kuttner & Wiesner, 1987). This mechanism may also be responsible for the low white blood cell counts in this study. It is also worth noting that the granulocyte count in our study was significantly higher than that reported by Lumsden et al. (1980) in horses, whereas no statistically significant differences were observed in the absolute lymphocyte counts, which were low.

### 4.2 Biochemical blood parameters of Calf Saiga antelope

Biochemical blood parameter levels are essential for defining the biochemical profiles, energy metabolism, metabolic disorders, liver function and bone abnormalities as a means of determining adaptation levels of animals (Swenson & Reece, 2006).

In this study, triglyceride and magnesium concentrations were significantly higher in males than in females (*p* < .05) as was immunoglobin activity (*p* < .01), whereas females exhibited higher HBDB activities (*p* < .01). Supatsak et al. (2016) noted higher immunoglobulin activities in male, whereas Helen and Lindsay (1972) reported lower immunoglobulin values for normal and ketonaemic sheep. Significantly higher cholesterol levels were measured in females relative to males (*p* < .01) in contrast to the findings reported by Asadi, Mohri, Adibmoradi, and Pourkabir (2006) Moreover, Guedon, Saumande, Dupron, and Desbals (1999) conducted a study on the beef cows and of found that cholesterol values decreased significantly at the end of pregnancy, reaching a minimum at parturition, after which they gradually increased for up to nine weeks post‐calving. Calcium, magnesium and inorganic phosphorus are three important minerals in animal blood, and they play an essential role as structural components of the body of an animal and in enzyme and hormone activities. These minerals contribute to bone formation, heart and other structures of an animal's body. In contrast, other studies have shown that insufficiency of phosphorous, calcium and magnesium might be the main causes of some diseases involving fatigue, pica, arthralgia and bone pain (Aytekin & Kalinbacak, 2008; Jain & Chopra, 1994). Concentrations of these three important minerals were normal in our study, especially magnesium, which normally shows significantly higher counts in these antelopes than in other animals.

Concentrations of albumin and their ratios in the plasma of Calf Saiga antelope did not differ from most reference values. Ranges of parameters were slightly wider than in the domestic horses. Total protein in the serum reflected good nutritional status, but total protein in this study fell within the reference range compared with Przewalski horse (Tomenendalova et al., 2014) and was lower when compared with the study done on the Equus Przewalski horse (Kuttner & Wiesner, 1987). Likewise, ranges of ions in the serum corresponded with reference limits for domestic horses.

However, the most profound differences surrounded glucose concentrations. High levels of glucose in blood will damage the organs and body of an animal and lead to many metabolic instabilities in the long‐term (Hollis, Boston, & Corley, 2007). The glucose values found in this study were considerably higher than those in a previous study conducted on the Equus Przewalski horses (Kuttner & Wiesner, 1987) and nearly twice as higher when compared with the median value for the domestic horses (Lumsden et al., 1980). However, glucose levels in our study fell but were still within the normal reference range. The higher glycaemia levels may have resulted from the stress of separation from the herd in addition to immobilization (Kuttner & Wiesner, 1987).

Furthermore, enzyme activities in the Calf Saiga antelope were higher than in domestic horses but not considerably different from the species‐specific data. It is therefore, clear that biochemical reference ranges from domestic or Przewalski horses are not suitable for the evaluation of haematological findings in the domestic horse, beef cow, growing goat and sheep. Triglyceride levels were similar, but concentrations were higher in beef cows (Guedon et al., 1999).

Creatinine has shown to be associated with muscular damage and is used as a welfare indicator; serum creatinine levels may serve as a useful marker in assessing and monitoring the nutritional status in animals (Mauro et al., 2014; Stefanie et al., 2017). Increases in creatinine kinase were associated with chronic low‐grade muscle damage (Aroch et al., 2010). Although creatinine concentrations in this study fell below the reference values, another study (Stefanie et al., 2017) conducted on the growing goat also reported that creatinine was also below the reference value. These activities may be due to specific‐species differences among animals. Urea is a nitrogen‐containing waste substance mainly distributed in blood and urine of animals. Concentrations of urea in this study were higher than the reference ranges but opposite to a study done on the sheep that have showed that urea levels were within the reference ranges but presented values at the higher end of the reference range (Giuseppe et al., 2006). The cholesterol levels found in this study were lower than reported by other researchers (Baronetzky‐Mercier, 1992; Lumsden & Mullen, 1978).

### 4.3 Mechanism affecting the haematological and biochemical parameters

#### 4.3.1 Haematological factors

The blood system remains sensitive to temperature changes and is an important indicator of haematological responses to stressors. Several factors, such as species, breed, sex, age, nutrition, diseases, physiological stage and seasonal variations can affect the pattern of haematological values (Bezerra et al., 2008). Stress has been proven to be mammalian body reactions to stimuli that disturb body homeostasis. These stimuli can be nutritional, biochemical, psychological or thermal. Heat stress requires more attention, considering that the environmental temperature variations have major effects on animal production (Leite et al., 2018; Nardone, Ronchi, Lacetera, Ranieri, & Bernabucci, 2010; Salama et al., 2014). These effects have been greater recently due to the advent of global warming and by the increase in exposing animal species worldwide to stressful conditions. High environmental temperatures in tropical and arid areas and low environmental temperatures in temperate areas can be lethal, and these temperatures challenge the capability of an animal to maintain the balance of its body (Silanikove, 2000a). The interaction between the animals and the environment and the capability of each species and breed to adapt are essential characteristics. Based on these characteristics, decisions must be made about the best system of raising and the best management strategy to be adopted to increase the production of the animals, including the Calf Saiga antelope (Mirkena et al., 2010). Climatic variations directly affect animals, including the Calf Saiga antelope, and causes changes in their physiology (Ribeiro et al., 2016). The impact of climate changes has promoted loss of many mammals and other animals (Salama et al., 2014), a fact that generates the need for detailed studies to better understand the mechanisms of animals to adapt to extreme temperature changes. The description of the production environment is important since each breed has distinct adaptability features. Changes in biological functions of the animals due to exposure to heat stress include physiological, hormonal, haematological and biochemical responses, which make the Calf Saiga antelope and other animals resistant capable of surviving in adverse environments. Among the hematological variables commonly assessed in studies of adaptability in ruminants are rectal temperature, heart rate, respiratory rate and surface temperature (Maria, Neila, Riccardo, & Roberto, 2018). Generally haematological variables in Calf Saiga antelope and other mammals change accordingly depending on season, age, sex, time of day, physiological stage, exercise, water consumption, food intake and digestion (Otoikhian, Orheruata, Imasuen, & Akporhuarho, 2009; Phulia, Upadhyay, Jindal, & Misra, 2010). In Calf Saiga antelope and other mammals as well, the skin is an important route for heat exchange between the body surface and environment. The blood flow to the skin is variable and modified as necessary in order to regulate rectal temperature. Both redirection of blood flow and vasodilation facilitate the dissipation of heat by sensible means, thus reducing the superficial temperature. The efficiency of heat loss through the skin depends on the temperature gradient between the animal body and the surrounding environment. The dissipation of heat in the animal's body by insensitive ways is used when the superficial temperature increase is caused by the redirection of blood flow to the body surface and by vasodilation (Habeeb, Marai, & Kamal, 1992). All these and many other factors can influence the haematological and biochemical parameters of the Calf Saiga antelope and other animals.

#### 4.3.2 Biochemical factors

The interpretation of biochemical profiles is complex due to both the mechanisms that control the blood level of various metabolites and the considerable variation in these levels promoted by several factors (Gomide et al., 2004). Among these factors, the authors can highlight the breed, age, physiological stage, diet and management of the animal and the climate. In addition, some studies have shown that in a high‐temperature environment, blood glucose and cholesterol levels decrease, which is an indicator of homeostatic failure. However, glucose in this study fell but was still within the reference range (Ocak & Guney, 2010; Ribeiro et al., 2016). The maintenance of stable blood glucose levels is regulated by the liver, extrahepatic tissues and hormones, namely insulin, glucagon, adrenaline, cortisol and thyroid hormones (Swenson & Reece, 2006). Furthermore, the blood plasma lipids are composed of three major groups: (a) cholesterol; (b) phospholipids and (c) triglycerides. Triglycerides are mobilized as a source of energy (Payne & Payne, 1987) when failures in glucose requirements occur, but the findings from this study imply that glucose levels of the Calf Saiga antelope were within normal levels. Heat stress had a greater effect on the total cholesterol levels, which may be due to the increase in fatty acid use for energy production as a consequence of the reduction in glucose concentration in animals undergoing heat stress (Mundim, Costa, Mumdim, Guimaraces, & Espindola, 2007). In this study, females were characterized by significantly high cholesterol levels (*p* < .01).

## 5 CONCLUSION

The reported results clearly show that the characteristics of haematological and biochemical parameters varied among Calf Saiga antelope, horses, beef cow, Catalonian donkeys (*E. asinus*) and sheep. Furthermore, biochemical parameters available in Calf Saiga antelope are not suitable for determination of haematological findings in the horses, beef cow, Catalonian donkeys (*E. asinus*) and sheep. Since the use of data reported for Saiga antelope by other researchers may differ, these variations could have an impact on research outcomes. Therefore, further studies are needed to determine the way in which specific variabilities influence the quality of measured parameters. In addition, researchers should not rely on data from one group of species to use in an analysis or to conduct an investigation in another species. Rather, further studies on this topic should collect the most appropriate parameters to research a particular animal. This study also explored various mechanisms for factors affecting biochemical and physiological parameters. As such, our study proposes that regardless of these factors, breed, breeding environment and climatic variables can trigger haematological, and biochemical variations. Subsequently, the result is a reduction in the amount of heat produced for maintenance of homeothermy. Furthermore, these changes have a substantial influence on determination and investigation of biochemical and haematological parameters of the Calf Saiga antelope. This study is unique with respect to the sample size and the number of animals that were analysed.

## CONFLICT OF INTEREST

The authors declare an absence of any conflicts of Interest.

## ETHICAL STATEMENT

All experimental procedures used in this study were approved by Animal Ethical and Welfare Committee of Gansu Endangered Animals Research Center, Gansu Province in September 2018. Approval No. AEWC‐GEARC‐2018006.
